# Analysis of T Cell Receptor Vβ Diversity in Peripheral CD4+ and CD8+ T Lymphocytes Obtained From Patients With Chronic Severe Hepatitis B

**DOI:** 10.5812/hepatmon.15900

**Published:** 2014-02-25

**Authors:** Ying Xiong, Yan Tan, Yu Guo Song

**Affiliations:** 1 Central Laboratory, First Affiliated Hospital, Jilin University, Changchun, China; 2 Life Science Research Center, Beihua University, Jilin, China; 3 Cancer Biotherapy Center, Jilin Province People’s Hospital, Changchun, China

**Keywords:** Hepatitis B, Genes, T-Cell Receptor, CD8-Positive T-Lymphocytes, Complementarity Determining Regions, Clonal expansion, Immunity, Cellular

## Abstract

**Background:**

The hepatitis B virus (HBV) antigen-induced cellular immune response plays an important role in HBV clearance. Changes in the diversity of complementarity determining region 3 (CDR3) and T-cell receptor (TCR) sequences are used to monitor the response of T cells to antigens.

**Objectives:**

The aim of the present study was to determine whether the TCR Vβ repertoire of patients with chronic severe hepatitis B (CSHB) undergoes increased stimulation, and to identify conserved motifs in specific TCR Vβ families.

**Patients and Methods:**

Peripheral blood mononuclear cells (PBMCs) from 18 patients with CSHB were sorted into CD4+ and CD8+ T subsets, using monoclonal antibody-coated magnetic beads. The TCR Vβ CDR3 was subsequently characterized using immune spectratyping. The TCR Vβ families exhibiting a CDR3 spectratype that underwent monoclonal expansion were sequenced.

**Results:**

The number of oligoclonal or monoclonal expansion TCR Vβ families detected in the analyzed CD8+ T cells was significantly higher than the number detected in CD4+ T cells. The CDR3 spectratype analysis showed predominant usage of TCR Vβ5, Vβ7, Vβ9, Vβ12, and Vβ18 families in CD8+ T cell subsets of CSHB patients. Furthermore, conserved amino acid motifs were found to be associated with the monoclonal expansion of CD8+ TCR Vβ families. In addition, JB1S1 and JB2S7 region genes were present at a high frequency.

**Conclusions:**

The CD4+ and CD8+ TCR Vβ gene families undergo clonal expansion in CSHB patients, and CD8+ T cells play a major role in the pathogenesis of CSHB. Moreover, the conserved motifs and limited use of joining region genes observed in the CSHB patients of this cohort indicated that similar antigenic epitopes are recognized.

## 1. Background

Hepatitis B virus (HBV) infection is a major health problem worldwide. While some individuals, who develop an acute HBV infection, can clear the virus and achieve life-long immunity, others develop a chronic HBV infection. The latter is associated with various clinical manifestations ranging from an asymptomatic carrier state, with normal liver histology, to severe and chronic liver disease, including cirrhosis and hepatocellular carcinoma (HCC) (1, 2). A mortality rate greater than 50% is reported for those who develop chronic severe hepatitis B (CSHB) (3, 4). 

The HBV antigen-induced cellular immune response plays an important role in HBV clearance (5). This response involves both major histocompatibility complex (MHC) class II–restricted CD4+ helper T cells and MHC class I–restricted CD8+ cytotoxic T lymphocytes. Correspondingly, the pathogenesis of CSHB has been shown to be related to significant increases in the levels of CD8+ and nonspecific T cells (6). While CD4+ T cells do not directly participate in viral clearance and tissue damage, it is hypothesized that they indirectly control HBV infection by facilitating the induction and maintenance of virus-specific B cells and the CD8+ T cell response (7).

T cells recognize antigens by binding T cell receptors (TCRs) present on the surface of lymphocytes. Approximately 95% of CD4+ and CD8+ T cells are composed of an ɑ- and β-chain, and the β-chain gene complex of the human TCR includes at least 57 variable (V) gene segments. These gene segments have been further divided into 24 TCR Vβ gene families. The hypervariable region of each TCR Vβ family, referred to as the complementarity-determining region 3 (CDR3), is formed by joining V-D-J segments. These recombination events then establish the diversity of the immune responses manifested by individuals.

The CDR3 is one of the three regions, which determine T cell antigen specificity. Therefore, changes in CDR3 diversity and TCR sequences are used to monitor the response of T cells to antigens. In particular, spectratyping is a widely used technique able to measure the TCR repertoire diversity based on variations in the lengths of the reverse transcriptase-PCR (RT-PCR) products generated (8). Thus, spectratyping represents an important tool for monitoring antigen-driven clonal expansion or depletion of T lymphocytes. Correspondingly, immune spectratyping has been widely used in the recent years to detect the clonal features of T cells and to analyze the TCR CDR3 gene (9, 10).

While accumulating evidence has suggested that clonal expansion of T cells plays a major role in the immune pathogenesis of patients with chronic hepatitis B (CHB), only a few studies have described the individual TCR clonal features of CD4+ and CD8+ T cell subsets for patients with CSHB (11). In this study, the CD4+ and CD8+ TCR Vβ repertoire from 18 CSHB patients were analyzed, using immunoscope spectratyping. It is hypothesized that characterization of the CDR3 spectratypes of the 24 Vβ families would provide valuable insight into the development of DNA vaccines and individualized immunotherapies for CSHB (12).

## 2. Objectives

The aim of the present study was to determine whether the TCR Vβ repertoire of patients with CSHB undergoes increased stimulation, and to identify conserved motifs in specific TCR Vβ families.

## 3. Patients and Methods

### 3.1. Subjects

Eighteen CSHB patients with positive results for HBV antigens for at least 12 mo were admitted to the Department of Infectious Disease (First Affiliated Hospital, Beihua University, Jilin, China) between March 2011 and May 2012, and were enrolled in this study. These patients included 14 males and four females, with a mean age of 32.5 ± 1.2 y (range: 18–50). Eight age-matched healthy control donors were also included in this study. Both patients and donors provided written informed consent. Peripheral blood samples were obtained from each patient and healthy control. The presence of human immunodeficiency virus (HIV), hepatitis A virus (HAV), hepatitis C virus (HCV), hepatitis D virus (HDV), and hepatitis E virus (HEV) were excluded by laboratory testing. In addition, patients with CSHB had serum alanine aminotransferase (ALT) levels greater than 40 IU/L, total bilirubin (TBIL) levels greater than 170 μmol/L, and plasma prothrombin activity (PTA) levels less than 40% (13). This study was conducted according to the guidelines of the Declaration of Helsinki, and the Beihua University Medical Ethics Committee approved all procedures involving human subjects.

### 3.2. Serological Markers and Biochemical Evaluation

Serum ALT and TBIL levels were detected using an automatic biochemical analyzer (HITACHI 7080, Khiyoda, Tokyo, Japan). Enzyme-linked immunosorbent assay (ELISA) detection kits (Jingmei Biotech, Shenzhen, China) were used to detect HBV markers: HBsAg, HBeAg, HBeAb, and HBcAb.

### 3.3. Quantification of Serum HBV DNA

A quantitative real-time fluorescence kit (Shenzhen PG Biotech, Shenzhen, China) was used to quantify serum HBV DNA levels. The lower limit of detection for this kit was 1000 viral genome copies/mL.

### 3.4. Isolation of CD4+ and CD8+ T lymphocytes

Peripheral blood mononuclear cells (PBMCs) were isolated from the heparinized whole blood samples collected from CSHB patients (n = 18) using Ficoll-Hypaque gradient centrifugation (GE healthcare-science AB, Uppsala, Sweden). The CD4+ and CD8+ T lymphocytes were purified from PBMCs using monoclonal antibody-coated magnetic beads according to the manufacturer’s instructions (Invitrogen Dynal AS, Oslo, Norway). Briefly, an appropriate volume of CD4+/CD8+ Dynabeads was added to each tube containing a PBMC sample. After 20 min at 4°C with gentle tilting and rotation, the tubes were placed in a magnetic rack for 2 min to isolate each supernatant. After the supernatants were discarded, the beads were washed three times. Using flow cytometry, the purity of each isolated cell population was found to be greater than 90% (Beckman Coulter XL, Brea, CA, The USA).

### 3.5. Extraction of RNA and Synthesis of cDNA

Total RNA was extracted from 1 × 105 to 5 × 105 CD4+ / CD8+ T lymphocytes using a total RNA extraction kit (Promega, Madison, WI, The USA), according to the manufacturer’s instructions. Purified RNA was subsequently quantified using spectrophotometry. To synthesize first-strand cDNA, 1 ug total RNA was combined with 250 uM oligo dT (Promega, Madison, WI, The USA), 200 U M-MLV reverse transcriptase (Promega, Madison, WI, The USA), and 250 uM of each deoxyribonucleoside triphosphate (dNTP) (Promega, Madison, WI) in a total volume of 20 uL. Synthesis was performed at 42°C for 60 min, and then the reaction was terminated at 72°C for 10 min.

### 3.6. PCR Amplification of CDR3 cDNA

Using a two-step PCR assay, CDR3 length within the TCR Vβ chain was analyzed. The first round of amplification included 0.5 uL (20 uM) each of forward Vβ and reverse Cβ primers previously described by Kou et al. (14), 0.5 uL (10 mM) dNTPs, 0.25 uL cDNA, 2.5 uL 10 × PCR buffer, and 1.25 U Taq polymerase for a total reaction volume of 25 uL in sterile H2O. These samples were then subjected to PCR amplification as follows: 3 min at 95°C, 35 cycles of 95°C for 45 s, 55°C for 45 s, and 72°C for 45 s, followed by a final extension step at 72°C for 5 min. The resulting product was then used as the template for a second round of PCR amplification. For the second step, the forward and reverse primers used (VβNS and CβNS, respectively) were designed to bind 3' to the first round Vβ-specific primer and 88 bp away from the CDR3 region, respectively. In addition, the reverse primer contained a nontemplate sequence, GTTTCTT, which was labeled with a blue fluorescent dye, 6-carboxyfluorescein (6-FAM). After an initial denaturing step at 95°C for 3 min, 30 cycles of 95°C for 30 s, 58°C for 30 s, and 72°C for 30 s were performed, followed by a final extension step at 72°C for 10 min. An aliquot of each PCR product (5 uL) was subsequently analyzed by agarose electrophoresis. After staining the gel with ethidium bromide, bands present were visualized under ultraviolet light.

### 3.7. Analysis of CDR3 Length by Spectratyping

Fluorescent PCR products and a size marker were mixed with formamide and denatured at 94°C for 2 min. Samples were then loaded onto a 6% acrylamide sequencing gel (National Diagnostic, Atlanta, GA, The USA) and sequenced using a 24-lane Applied Biosystems model 373 DNA sequencer (Applied Biosystems, Foster City, CA, The USA). After 6 h, the data were analyzed and quantified using the ABIPRISM GeneScan analysis software (Applied Biosystems, Foster City, CA, The USA). The relative intensity (RI) of each product was used to generate a curve for each Vβ family with relative fluorescence intensity calculated as follows: 10 × (clonal peak area) / (total peak area). Monoclonal versus oligoclonal T cell expansion was distinguished according to the presence of a single peak with an RI greater than 35% or two peaks, each with an RI greater than 25%, respectively.

### 3.8. Molecular Cloning and Sequencing of CDR3 Segments

First-round PCR products were amplified using forward and reverse Vβ PCR primers, which were not fluorescently labeled. The PCR products were then separated using agarose gel electrophoresis and individual bands were purified using a gel extraction kit (Qiagen, Valencia, CA, The USA) according to the manufacturer’s recommendations. The resulting DNA was directly cloned into TA cloning vector PCR 2.1 (Invitrogen, Carlsbad, CA, The USA), sequenced using fluorescent dideoxy terminators, and analyzed using the Applied Biosystems model 337A automated sequencer. The amino acid sequence of the CDR3 region was analyzed using DNAMAN software version 1.0 (Lynnon Biosoft, Pointe-Claire, Quebec, Canada).

### 3.9. Statistical Analysis

Statistical analyses were performed using SPSS 16.0 software (SPSS Inc., Chicago, IL, The USA). Differences between CD4+ and CD8+ T cell datasets were examined using the χ^2^ -test. A P value < 0.05 was considered statistically significant. 

## 4. Results

### 4.1. CD4+ and CD8+ T cell Clonality in CSHB Patients 

**Figure 1.**
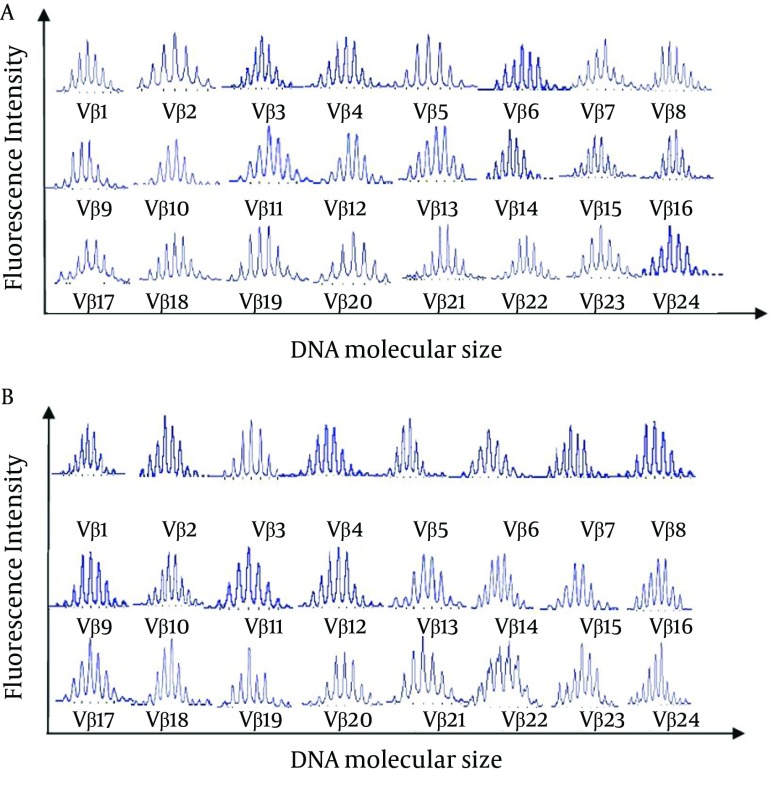
CDR3 Spectratypes of 24 TCR Vβ Families Detected in (A) CD4+ T Cells and (B) CD8 + T Cells Obtained From a Healthy Control (No. 1)

**Figure 2.**
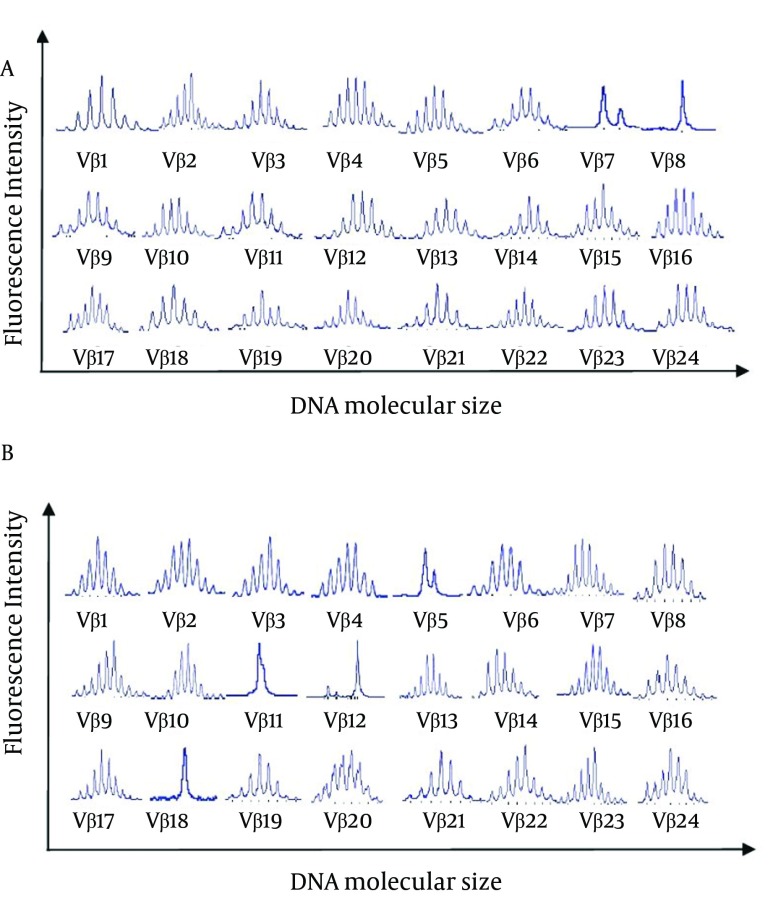
CDR3 Spectratypes of 24 TCR Vβ Families Detected in (A) CD4+ T Cells and (B) CD8+ T Cells Obtained from a CSHB Patient (No. 6)

The CDR3 spectratypes determined for the 18 CSHB patients and eight healthy controls were compared. All of the healthy controls exhibited normally diversified TCR repertoires, with the TCR Vβ gene sequences having a Gaussian distribution of approximately eight peaks. The CD4+ and CD8+ TCR CDR3 spectratype profiles obtained for a healthy control are shown in Figure 1. For the CSHB patients, monoclonal or oligoclonal expansion was detected in 18/18 CD8+ and 16/18 CD4+ samples (Table 1). Furthermore, the number of monoclonal and oligoclonal TCR Vβ families in the CD8+ T cell subsets assayed was higher than that of the CD4+ T cell subsets (P < 0.01). Conversely, the number of normal TCR Vβ family expansions that occurred in CD4+ T cell subsets was higher than that of the CD8+ T cell subsets (2 vs. 0, respectively) (P < 0.05). An analysis of T cell clonality among the 18 CSHB patients also identified certain TCR Vβ families that were more prevalent, including: Vβ5 (27.8%), Vβ7 (44.4%), Vβ9 (27.8%), Vβ12 (50.0%), and Vβ18 (27.8%) (Table 2). The CD4+ and CD8+ TCR CDR3 spectratype profiles obtained for a representative patient are shown in Figure 2. 

**Table 1.** Oligoclonal or Monoclonal TCR Vβ Families of CD4+/CD8+ T Cell Subsets Detected for 18 CSHB Patients

Patients	CD4+ T Lymphocytes (n = 25 ^a^)		CD8+ T Lymphocytes (n = 49 ^a, b^)	
	Oligoclone	Monoclone	Oligoclone	Monoclone
**1**	9	12, 21	None	7, 9, 18
**2**	None	15	None	11, 16
**3**	9	None	8	5, 7, 12
**4**	4	8	7	9, 12, 17
**5**	None	12, 18	None	12, 15
**6**	7	8	5	11, 12, 18
**7**	3	None	12	None
**8**	2	7, 12	None	7, 12
**9**	None	21	2	8, 12
**10**	9	None	None	7, 21
**11**	11	None	4	5, 9
**12**	5	None	5	7, 18
**13**	None	5	20	12
**14**	None	None	4, 5	9, 21
**15**	None	9, 22	2	9
**16**	8	11	6	12, 18
**17**	None	None	11	7
**18**	18	None	7	18, 21

^a^ Indicates the total number of monoclonal or oligoclonal TCR Vβ families detected.

^b^ The rate of monoclonal or oligoclonal expansion of TCR Vβ families in CD8+ T cells was significantly higher than that for the CD4+ T cells (P < 0.01, by χ^2^test).

**Table 2.** The Frequency of Oligoclonal and Monoclonal TCR Vβ Families in CD4+ and CD8+ T Cell Subsets Obtained from 18 CSHB Patients ^a^

TCR Vβ Families	CD4+ T Cell Incidence, (n = 25^b^)	CD8+ T Cell Incidence, (n = 49^c^)
**1**	0	0
**2**	1 (6.25)	2 (11.1)
**3**	1 (6.25)	0
**4**	1 (6.25)	2 (11.1)
**5**	2 (12.5)	5 (27.8)
**6**	0	1 (5.56)
**7**	2 (12.5)	8 (44.4)
**8**	3 (18.8)	2 (11.1)
**9**	4 (25.0)	5 (27.8)
**10**	0	0
**11**	2 (12.5)	3 (16.7)
**12**	3 (18.8)	9 (50.0)
**13**	0	0
**14**	0	0
**15**	1 (6.25)	1 (5.56)
**16**	0	1 (5.56)
**17**	0	1 (5.56)
**18**	2 (12.5)	5 (27.8)
**19**	0	0
**20**	0	1 (5.56)
**21**	2 (12.5)	3 (16.7)
**22**	1 (6.25)	0
**23**	0	0
**24**	0	0

^a^ The average rate of monoclonal or oligoclonal expansion by TCR Vβ families in the CD8+ T cell subsets was significantly higher than that for the CD4+T cell subsets.

^b^ Average ratio for a case: 1.56.

^c^ Average ratio for a case: 2.72.

### 4.2. Sequence Analysis of the TCR Vβ Chain CDR3 Region 

Using Genescan, the PCR products for the TCR Vβ CDR3 amplified from CD8+ T cells showing monoclonal expansion were cloned, sequenced, and translated. An analysis of these sequences detected a high frequency of the J gene segments, Jβ1S1 and Jβ2S7. In addition, three amino acid motifs, GSF, LF, and GS were found to be conserved (Table 3). In contrast, no amino acid motifs were found to be conserved in the TCR CDR3 region of the analyzed CD4+ T cells.

**Table 3.** Partial Sequences of the CD8+ TCR CDR3 β-chain Showing Evidence of Monoclonal Expansion

Patients	BV ^a^	3’Vβ ^a^	N-D-N ^a^	5’Jβ ^a^
**1 (CD8)**	7	CASS	CPAGSFF ^b^	TCRBJ1S1
**3 (CD8)**	7	CASS	TGSFFTAQ	TCRBJ2S7
**8 (CD8)**	7	CASS	DKGSFG	TCRBJ2S7
**10 (CD8)**	7	CASS	EAGAAGSF	TCRBJ2S2
**12 (CD8)**	7	CASS	SSPYGSFF	TCRBJ1S2
**17 (CD8)**	7	CASS	TGSFGP	TCRBJ1S1
**1 (CD8)**	9	CASS	GVFDLFG	TCRBJ1S1
**4 (CD8)**	9	CASS	MILFSQBR	TCRBJ1S1
**11 (CD8)**	9	CASS	DDVRFG	TCRBJ1S1
**14 (CD8)**	9	CASS	PGTRWNED	TCRBJ2S7
**15 (CD8)**	9	CASS	ZZDSBLFDD	TCRBJ1S1
**3 (CD8)**	12	CASS	SGRYSSYD	TCRBJ1S1
**4 (CD8)**	12	CASS	LFASERGP	TCRBJ1S1
**5 (CD8)**	12	CASS	ESGLFERT	TCRBJ2S7
**6 (CD8)**	12	CASS	SRLFGETFQ	TCRBJ1S1
**8 (CD8)**	12	CASS	PPGSNQB	TCRBJ1S1
**9 (CD8)**	12	CASS	PGIFGLF	TCRBJ2S7
**13 (CD8)**	12	CASS	VSGSTVRT	TCRBJ1S1
**16 (CD8)**	12	CASS	RDGSFYGTG	TCRBJ1S1

^a^ BV, TCR Vβ family; Jβ: joining region of TCR Vβ CDR3; N-D-N, diversity region of TCR Vβ CDR3; Vβ, variable region of TCR Vβ CDR3.

^b^ underlined words show the conserved motif of TCR CDR3.

## 5. Discussion

Immune spectratyping has been used to assess TCR diversity for both healthy and disease states. Typically, the fragment lengths generated would have a Gaussian-like distribution. However, if clonal T cell expansion or a severe cytoreduction has occurred, the proportional distribution of different CDR3 lengths is affected. In previous studies, spectratyping has facilitated the identification of tumor-, pathogen-, or allo-antigen-driven oligoclonal expansions within the TCR repertoire (15-17).

In the present study, the CDR3 in CD4+ T cells and CD8+ T cells were spectratyped separately. This is in contrast with previous studies, where TCR CDR3 size diversity was analyzed in a combined population of CD4+ and CD8+ T cells obtained from patients with CHB (18, 19).

In the present study, both monoclonal and oligoclonal expansion were detected for the CD8+ TCR Vβ families of all the 18 patients examined. Moreover, the monoclonal expansion of CD8+ TCR Vβ5, Vβ7, Vβ9, Vβ12, and Vβ18 families were found to be more prevalent than other TCR Vβ families. In contrast, the study of Yang et al. (11) to detect the oligoclonal expansion of T cells in CSHB patients demonstrated that TCR Vβ7 and Vβ11 were expressed more frequently compared to the other members of the TCR Vβ family in PBMCs, and in CD8+ and CD8- subsets. It is possible that differences in the HLA types of the patients analyzed, as well as the HBV genotypes, HBV variants, and the detection methods used, may account for the differences observed between these studies. 

Exogenous (plasma) HBV antigens can be processed by macrophage and presented to CD4+ T cells. This can enhance the synthesis of cytokines, which augment T-cell proliferation, thereby increasing the number of HLA class I molecules present on hepatocytes and decreasing viral replication. In the present study, monoclonal and oligoclonal expansion of CD4+ T cells was detected in 16/18 patients. Furthermore, the average rate of monoclonal and oligoclonal expansion in CD4+ and CD8+ T cell subsets were 1.56 and 2.72, respectively. The number of monoclonal or oligoclonal expansion TCR Vβ families in CD8+ T cells was significantly higher than that in CD4+ T cells (P < 0.01), and this is consistent with the results of previous studies where proliferating HBV antigen-specific T cells were found to be CD8+ type (20). For a better understanding of the nature of T cells in disease presentation associated with HBV infection, monoclonal expansion of CD4+/CD8+ TCR Vβ CDR3 was sequenced. As a result, highly conserved amino acid motifs were identified in the CD8+ TCR CDR3, and a higher frequency of BJ1S1 and BJ2S7 was identified in the proximal region of TCR CDR3. Several studies have shown that single viral or bacterial epitopes can induce T cell clones, including different TCR CDR3 sequences. It is possible that the pattern of CDR3 size distribution for TCR Vβ in different CSHB patients may provide information regarding HBV antigens, especially if a certain CDR3 indicates that a particular peptide antigen of HBV was present. However, due to the diversity of HBV and human leukocyte antigens, T cells related to HBV were found to be different in CHB patients (21, 22).

T cells can identify several types of HBV antigens that have completely different structures. Therefore, it is reasonable to speculate that conserved sequences identified in an analysis of CDR3 sequences from a large number of CSHB patients may represent TCRs specific for a particular HBV antigen. For the CDR3 sequences analyzed from the 18 CSHB patients in the present study, three conserved amino acid motifs were identified, including GSF, LF, and GS. These motifs were present in the β-chains and may be involved in binding to a HBV antigen that is common to a number of CSHB patients. More studies are needed to further characterize the association between these conserved motifs and the corresponding antigen(s) present in CSHB patients, with the overall goal of elucidating the function of various TCR Vβ gene families in the progression of CSHB.

In summary, the results of the present study provided evidence that peripheral CD4+ and CD8+ T cells from CSHB patients have undergone clonal expansion. The detection of pathogenic T cells in CSHB patients also provides useful insight regarding the pathogenesis and status of this disease. It is expected that the results of the present study would facilitate the development of DNA vaccines and individualized immunotherapies for CHSB, in combination with future studies, to characterize the conserved motifs identified.
